# Identifying Eligibility for Specialist Intervention in COPD from UK Primary Care Data: A “Treatable Traits” Approach

**DOI:** 10.2147/COPD.S502865

**Published:** 2025-08-06

**Authors:** Thomas J C Ward, Catherine John, Alexander T Williams, Chiara Batini, Neil J Greening, Martin D Tobin, Michael C Steiner

**Affiliations:** 1 Department of Respiratory Sciences, University of Leicester, Leicester, UK; 2 University Hospitals of Leicester, Leicester, UK; 3 Institute for Lung Health, National Institute for Health Research Leicester Biomedical Research Centre – Respiratory Glenfield Hospital, Leicester, UK; 4 Department of Population Health Sciences, University of Leicester, Leicester, UK

**Keywords:** integrated care, chronic respiratory disease, treatable traits

## Abstract

**Background:**

Specialist intervention in COPD is often reactive, resulting in inequalities in the provision of care. A proactive approach, in which individuals with modifiable disease are identified from primary care records, may help to tackle this inequality in access.

**Aim:**

To estimate the prevalence of “treatable traits” in COPD in a primary care research database and to assess health service usage.

**Methods:**

We performed a secondary analysis of individuals with either 1) a primary care diagnosis of COPD or 2) obstructive spirometry and history of ever smoking in a large observational study recruiting individuals aged 40–69 years old in Leicestershire, UK. Spirometry, height, weight and smoking history were collected prospectively and linked to individuals’ primary care records. “Treatable traits” were identified from primary care records (frequent exacerbations, current smoking, low body mass index, respiratory failure, severe breathlessness, potential suitability for lung volume reduction or psychological comorbidity). Differences in demographics and health usage between those with and without “treatable traits” were assessed.

**Results:**

In total, of the 347 individuals with COPD, 186 had at least one “treatable trait”. Compared to those without treatable traits, individuals with treatable traits were younger (61 vs 64 years, p<0.001), had more severe airflow obstruction (FEV_1_ 86% vs 94% predicted, p=0.002), higher eosinophil count (0.32 vs 0.27 cells/μL, p=0.04) and were more socioeconomically deprived (UK Indices of Multiple Deprivation decile 4.3 vs 5.8, p<0.001). Individuals with treatable traits had a higher annual primary care health usage (47 vs 30 visits per year, p=0.001). Referrals rates to specialist respiratory services were low in both groups.

**Conclusion:**

Treatable traits are common in COPD and can be identified from routinely collected primary care data. Treatable traits are associated with younger age and greater deprivation. These individuals pose a significant burden to primary care yet are rarely referred to specialist respiratory services.

## Introduction

Most people with COPD are managed in primary care with specialist intervention usually reactive, yet people from underserved communities may not access the specialist care they are eligible for, resulting in inequalities in the provision of care.^1^ There is evidence that specialist led integrated COPD care is likely to be superior to usual care^2^,^3^ leading to better adherence to guidelines and improvements in health-related quality of life. However, given the high prevalence of COPD with an estimated 4.9% of the UK population affected,^4^ specialist led care will need to be targeted to be deliverable. Specialist review in COPD is likely to be more efficiently delivered if targeted at people with certain disease phenotypes, such as severe breathlessness or frequent exacerbations, who may be eligible for specific specialist interventions.^5^

Routinely collected primary care data may provide an opportunity to deliver a targeted approach to COPD management.^6^ It may be possible to identify individuals from their primary care record that have modifiable disease phenotypes likely to benefit from specialist intervention. Such an approach may help to tackle inequality in access as referral could be matched to clinical need rather than the ability to access care and as such may improve outcomes. However, before this approach can be considered, we need to understand the feasibility of identifying such “treatable traits” from routinely collected clinical data.

We aimed to estimate the local prevalence of COPD with specific treatable disease traits likely to benefit from specialist intervention in a primary care research database and to investigate the health service usage of this population. We hypothesised that treatable traits are identifiable in primary care data and associated with higher healthcare usage.

## Methods

This was a longitudinal analysis of participants in EXCEED (Extended Cohort for E-health, Environment and DNA), a large observational study recruiting individuals aged 40–69 years old in Leicester City, Leicestershire, and Rutland, UK, since 2013^7^ which was approved by the East Midlands Regional Ethics Committee (13/EM/0226). All participants consented for their data (including primary care data) to be used for future research projects approved by the EXCEED data access committee. All participants were informed about the purpose of the study, in accordance with the Declaration of Helsinki. Inclusion criteria for EXCEED were patients registered with participating primary care practices aged between 40 and 69 years and people registered with smoking cessation services aged between 30 and 69 years. Exclusion criteria were those receiving palliative care, those with learning disabilities or dementia and those whose records indicated they had declined consent for record sharing for research. Spirometry, height, weight, smoking history, ethnicity and index of multiple deprivation decile (IMD) were collected prospectively and linked to individuals’ primary care records. A list of specific primary care codes for each treatable trait was extracted for consenting participants (Supplementary Tables 1–13).

Our analysis was restricted to individuals with either 1) a primary care diagnosis of COPD or 2) obstructive spirometry (FEV_1_/FVC <0.7) at study entry with individuals reporting never smoking excluded. COPD with “treatable traits” was defined as meeting one of the following criteria:^8^ history of frequent exacerbations of COPD (>2 in a single year); history of 2 or more hospitalisations for exacerbations of COPD (any time period); current smokers; low body mass index (<22 kg/m^2^); primary care code of weight loss; respiratory failure (including use of home oxygen, use of non-invasive ventilation (NIV)); severe breathlessness (Medical Research Council dyspnoea score 3 or worse); potential candidate for lung volume reduction therapy (FEV_1_ < 50% predicted); and psychological comorbidity. Outcomes were average annual primary care usage over the 5 years prior to recruitment (based on total number of primary care codes per year), referrals to pulmonary rehabilitation, number of hospitalisations, specialist respiratory referrals and referral to smoking cessation services. A sensitivity analysis was conducted restricted to individuals with a primary care code of COPD.

Primary care codes in the 10 years preceding recruitment were considered. Comparisons between groups were conducted using t-tests, chi-squared tests or fisher exact tests as appropriate. Correlation between number of traits and healthcare usage was performed using Pearson correlation. All analyses were pairwise with no corrections applied. Missing data was not imputed. Statistical analysis was conducted in R version 4.2.2.

## Results

**Figure 1:**
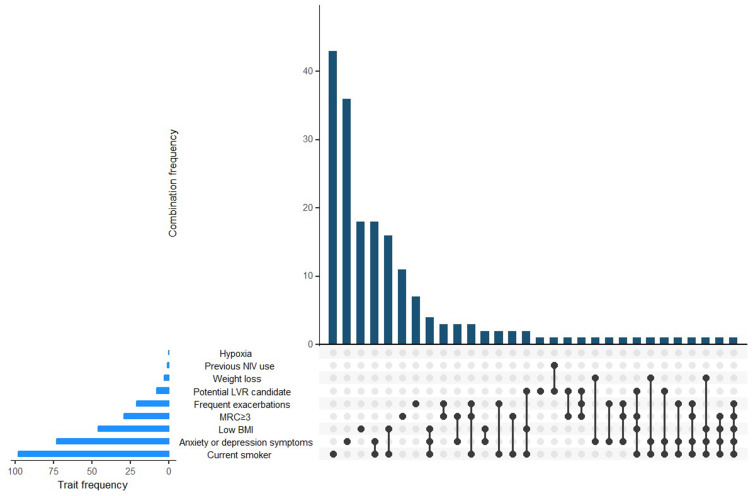
Upset plot showing frequency of treatable traits and treatable trait combinations. Each vertical bar represents a unique combination of traits shared among patients, with bar height corresponding to frequency. Individual trait frequencies are displayed as horizontal bars on the left, representing the total number of patients exhibiting each trait.

From 550 participants in EXCEED with FEV_1_/FVC <0.7 or a primary care code of COPD, 203 participants who reported never smoking (less than 100 cigarettes in lifetime) were removed. Of the remaining 347 individuals, 186 had at least one treatable trait (Supplementary Figure 1). The frequency of treatable traits was as follows: 21 individuals had more than two exacerbations in a year, 98 were current smokers, <5 had a primary care code for weight loss, 46 had BMI <22 kg/m^2^, <5 used oxygen therapy or NIV, 10 had FEV_1_ <50% predicted, 30 had MRC dyspnoea scale ≥3, 75 had a code for anxiety or depression (Figure 1).

**Figure 2:**
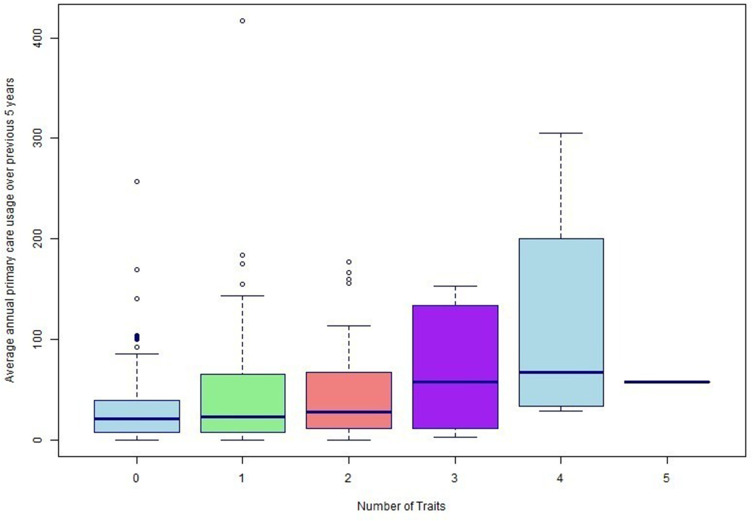
Box plot of average annual primary care usage split by total number of treatable traits.

Individuals with treatable traits were younger, had more severe airflow obstruction, higher eosinophil count and were more socioeconomically deprived compared to those without treatable traits (Table 1). Though statistically significant, the absolute difference in eosinophils was small. Individuals with treatable traits had a higher annual primary care health usage (30.3 vs 46.9 codes per year, p=0.001). There was a positive correlation between an individual’s total number of disease traits and average annual primary care health usage (r=0.23, p=0.007, Figure 2). In a sensitivity analysis restricted to individuals with a primary care code of COPD (n=93), trait frequencies were generally similar although severe breathlessness (MRC ≥ 3) and frequent exacerbations were more common in those with a primary care coded COPD diagnosis (Supplementary Figure 2). Individuals with treatable traits referred to specialist respiratory services were older (63 vs 60 years, p=0.04), had higher BMI (30 vs 26 kg/m^2^, p=0.03), had higher average annual primary care usage (92 vs 40 codes per year, p=0.01) and a greater proportion had severe breathlessness (MRC≥3 in 52% vs 10%, p<0.001) compared to those not referred.

**Table 1:** Comparison Between Individuals with and without Treatable COPD Traits

	COPD without Treatable Traits (n=161)	COPD with Treatable Traits (n=186)	p (Difference Between Groups)
Gender (% male)	54%	40%	0.014*
Smoking pack years	28 ± 24	28 ± 22	0.838
Proportion of current smokers	0%	53%	NA
Age (years)	64 ± 6	61 ± 7	<0.001*
Index of multiple deprivation (IMD) decile (1 = most deprived)	5.8 ± 2.7	4.3 ± 2.8	<0.001*
Ethnicity (% White)	96%	95%	0.808
FEV_1_ (%predicted)	94 ± 19	86 ± 22	0.002*
Eosinophils (cells/μL)	0.27 ± 0.16	0.32 ± 0.28	0.040*
BMI (kg/m^2^)	28 ± 4.0	27 ± 6.4	0.005*
Average annual primary care usage in previous 5 years (including prescription visits)	30 ± 34	47 ± 57	0.001*
Number referred to PR in previous 10 years	0	10 (5%)	0.002*
Number referred to smoking cessation services in previous 10 years	≤5 (≤3%)	11 (6%)	0.045*
Number referred to respiratory outpatient or community respiratory team in previous 10 years	≤5 (≤3%)	7 (4%)	0.073
Number hospitalised in previous 10 years (any reason)	8 (5%)	22 (12%)	0.038*

**Note**: We have used ≤5 to represent censored data for privacy. *p<0.05.

**Abbreviations**: IMD, Index of multiple deprivation; FEV_1_, Forced expiratory volume in 1 second; BMI, body mass index; PR, pulmonary rehabilitation.

## Discussion

This analysis of UK primary care records demonstrates that proactively identifying individuals with COPD and treatable traits likely to benefit from specialist intervention from routinely collected primary care data is a realistic possibility. If scalable, this approach allows proactive identification of the presence of “treatable traits”, including eligibility for evidence-based specialist interventions, and may reduce inequalities in healthcare access. Individuals with treatable traits appear to have higher primary and emergency care usage than individuals without treatable traits and therefore providing targeted specialist intervention for these individuals may also provide system benefits by reducing unscheduled healthcare usage.

There is increasing acknowledgement of the importance of integrated respiratory services and an increasing number of clinical trials employing different approaches to case finding and management of airway disease in primary care.^2^,^3^ Whilst the concept of “treatable traits” in COPD is well established in tertiary care,^9^ as yet there are no published clinical trials employing this approach in a primary care setting.^5^ Our results suggest that the use of routinely collected clinical data may provide an opportunity for such a “treatable trait” approach in primary care. Whilst there is likely to be a degree of miscoding of “treatable traits” in primary care records, these results show that this approach may still be able to identify individuals with different characteristics and increased health usage. This may be operationalised through the use of automated electronic record alerts for treatable traits during COPD reviews.

Limitations of this analysis were that data were obtained from a research setting and the sample may not be entirely representative, particularly as the study population was predominantly White British despite a large local South Asian community. Individuals over 70 years old were excluded, and therefore the results may not be applicable to older individuals with COPD. COPD was defined by airflow obstruction and smoking rather than clinical assessment; however, this may be a more useful pragmatic approach for use in primary care. We chose to limit our analysis to individuals with a previous history of smoking to differentiate COPD from asthma as a cause of obstructive spirometry. Other causes of COPD such as biomass exposure are unlikely in our predominantly White British population but acknowledge this as a limitation. Some referrals may have been missed due to coding errors. We have used total healthcare usage as a crude measure but appreciate this limits the conclusions we can make on the effect of treatable traits on routine and unscheduled healthcare usage.

Individuals with COPD and treatable traits that may benefit from specialist intervention are common in primary care and can be identified from routinely collected health data. Treatable traits are associated with younger age and greater deprivation. These individuals pose a significant burden to primary care yet are rarely referred to specialist respiratory services. Proactive identification of those with “treatable traits” has the potential to improve outcomes for people with COPD and reduce health inequalities. This analysis requires replication in larger multi-ethnic primary care databases and supports the development of a randomised controlled trial to test a proactive, primary care, treatable trait-based approach to COPD care.

## Data Availability

We welcome requests for collaboration and data access via Catherine John (exceed@leicester.ac.uk).
